# Insect Assemblage and Insect–Plant Relationships in a Cultivated Guayule (*Parthenium argentatum* A. Gray) Plot in Spain

**DOI:** 10.3390/insects16080808

**Published:** 2025-08-04

**Authors:** Eduardo Jarillo, Guayente Latorre, Enrique Fernández-Carrillo, Sara Rodrigo-Gómez, José Luis Yela, Manuel Carmona

**Affiliations:** 1 Food Quality Research Group, Institute for Regional Development (IDR), Universidad de Castilla-La Mancha, Campus Universitario s/n, 02071 Albacete, Spain; eduardo.jarillo@uclm.es; 2 Instituto Técnico Agronómico Provincial de Albacete (ITAP), Parque Empresarial Campollano, 2a Avenida. N° 61, 02007 Albacete, Spain; guayente.latorre@uclm.es; 3 Centro de Investigación Agroambiental “El Chaparrillo”-CIAG (IRIAF), 13071 Ciudad Real, Spain; efernandezc@jccm.es; 4 Junta de Comunidades de Castilla-La Mancha, 13270 Ciudad Real, Spain; srodrigog@jccm.es; 5 DITEG Research Group, Facultad de Ciencias Ambientales y Bioquímica, Universidad de Castilla-La Mancha, Campus Tecnológico de la Fábrica de Armas, 45004 Toledo, Spain; joseluis.yela@uclm.es

**Keywords:** acclimatation, guayule, insect assemblage, insect–plant relationships, plant phenology, trophic role

## Abstract

This study aims to characterize for the first time the insect assemblage associated with sown, introduced guayule (*Parthenium argentatum* A. Gray, Asteraceae) in Castilla-La Mancha, Spain, and identify potential relationships with the crop. Insect sampling was conducted using nets and pan traps during spring and early summer, coinciding with the flowering period of the plant. A total of 352 insect species/morphospecies across 12 orders were identified. Diptera, Coleoptera, Hemiptera, and Hymenoptera were the most species-rich and abundant orders. Within these orders, Muscidae, Syrphidae, Tenebrionidae, Dermestidae, Miridae, Halictidae, and Apidae were the most numerous families. Guayule flowering intensity increased gradually until mid-June, aligning with the peak activity of pollinating Diptera. The majority of the identified insects (74.4%) were potential pollinators, while nearly 50% were detritivores and approximately 30% were herbivorous. The similarity in insect families and functional roles observed in this study to previous studies in the USA and Mexico suggest that guayule may serve as a similar trophic resource for insects in Spain, despite being a non-native species.

## Simple Summary

This study explores the types of insects found on guayule plants grown in Castilla-La Mancha, Spain, to understand how they interact with the crop. Guayule is a plant native to North America that is being introduced to new regions. In spring and early summer, during the flowering season of the plant, insects were collected with nets and traps. A total of 352 different insect species were identified, which are grouped into 12 major groups. Flies, beetles, bugs, and wasps were the most common. Many of the insects were pollinators, i.e., they helped the plant to reproduce by transporting pollen, while others fed on plants or helped decompose dead material. Guayule flowering increased steadily until mid-June, coinciding with the peak of pollinator activity. Approximately three out of the four insects found were able to act as pollinators, demonstrating that guayule supports a wide range of useful insects. These results suggest that guayule can benefit biodiversity in areas where it is grown and could play a positive role in sustainable agriculture. The results are similar to those of studies in other countries, which indicate that guayule attracts the same types of useful insects regardless of where it is planted.

## 1. Introduction

Guayule (*Parthenium argentatum* A. Gray), a perennial Asteraceae shrub native to northern Mexico and southwestern USA, offers a sustainable alternative for the production of natural rubber, which accumulates within its stems and roots [1]. The successful industrial cultivation of guayule requires a comprehensive approach that addresses various factors, such as production optimization, including mechanized planting and harvesting, and efficient rubber extraction techniques; agronomic management, including effective cultivation practices to maximize yield and quality; acclimatization, adapting the plant to diverse growing environments; and environmental sustainability, minimizing potential environmental impacts [2,3]. The historical cultivation of guayule in Spain between 1940 and 1960 demonstrated its stability as a crop and its low invasive potential [4]. Despite the cultivation of nearly 4000 hectares during this period [5], very few isolated plants remain today. To assess the potential environmental impact of reintroducing guayule, it is crucial to study its interaction with native insect fauna.

The scientific understanding of the arthropod communities associated with cultivated guayule, particularly outside its native range, is limited. Despite the fact that guayule resin contains a high concentration of sesquiterpenes, which have antifeedant activity against certain insects [6,7], a wide variety of insect fauna have been observed on this crop [8,9]. A comprehensive study from the 1980s identified 107 species across 13 orders and 60 families (collected over four years) in guayule plantations at the Texas A&M University Research Station [9]. Among these, 56% were herbivorous, although not necessarily harmful to guayule, 36% were predatory, 6% were parasitic, 1% were detritivores, and 1% were omnivorous. Historically, research on insect pests on guayule has primarily focused on species posing a direct threat to the plant, whether in open cultivation, nurseries, or greenhouses [10]. An early report highlighted scale insects of the genera *Ceroputo* sp., *Orthezia* sp., and *Targionia* sp. infesting the roots of wild and cultivated plants in Mexico [11]. Decades later, two *Targionia* species continued to cause issues in Mexican production areas, alongside other pests such as *Lecaniodiaspis* sp. species and *Tachardiella cornuta*, which significantly damaged branches and hindered plant growth [12].

Guayule nurseries, with their crowded conditions, high temperatures, and abundant water, are particularly susceptible to arthropod pests [8,13]. One prominent example is the mite, *Aceria parthenii*, which exhibits a strong preference for guayule hybrid seedlings [14]. Among the most abundant pests in three monitored nurseries were leafhoppers, wooly bear caterpillars, cucumber beetles (*Diabrotica undecimpunctata*), and *Lygus* bugs (Hemiptera; Miridae), with the latter being especially prevalent [8]. *Lygus* bugs pose a serious threat to guayule seed viability. *Lygus hesperus* lays eggs in flower heads, and both nymphs and adults feed on developing seeds, causing embryo collapse [15]. When seeds are unavailable, these bugs target growing stem tips, causing physical damage and injecting a toxic substance that kills the terminal meristem [15,16]. Additionally, wireworms of the genus *Limonius* spp. can inflict substantial root damage, which resulted in the death of 14% of nursery plants [10]. In the same period (the 1950s), greenhouse pests primarily included the two-spotted spider mite (*Tetranychus bimaculatus*), various aphids (*Myzus persicae* and *Aphis gossypii*), the greenhouse whitefly (*Trialeurodes vaporarioum*), and a leaf miner (*Phytomyza atricomis*) [10].

In industrial guayule plantations, grasshoppers pose the most significant threat. Species of the genus *Melanoplus* sp. and *Oedaleonotus enigma* (spur-throated grasshopper) in California can damage and kill plants by stripping bark from branches [10]. Additionally, in Zacatecas, Mexico, *Taeniopoda eques* (western horse lubber grasshopper) was found to feed in large numbers (about 100 individuals per plant) on the reproductive structures [17]. While this may not directly impact rubber production as severely as a lepidopteran pest such as *Eumeta hardenbergi*, which damages bark and leaves, reducing production by 100 kg/ha [18], it could negatively affect the nectar-feeding entomofauna and wider native food chains [17].

The problems posed by arthropods to guayule extend beyond the crop fields. Various beetle species including those from Curculionidae, Ptinidae, and Buprestidae families have been observed feeding on stored, baled shrubs [12].

While less extensively documented, guayule also hosts beneficial arthropods. For example, honeybees play a crucial role in pollination [19]. Additionally, the mymarid wasp *Anaphes ovijentatus* parasitizes *Lygus* species, a major potential pest of guayule [20,21,22]. Furthermore, predatory arthropods such as spiders, nabids, big-eyed bugs, lady beetles, and assassin bugs (Reduviidae) feed on Hemiptera and Homoptera [9], contributing to pest control.

Based on a preliminary trial conducted in 2022 in which monthly samplings were carried out for three months (July, August, and September) in guayule fields in Santa Cruz de la Zarza [23], the present study investigates the interplay between insects and guayule plants grown outside their native range for the first time. In addition to being the first study of its kind in Europe, this research also provides a long-overdue update to the entomological knowledge of guayule, much of which has not been revisited since the mid-20th century. The main aim was to characterize the insect species and functional groups present in Spanish guayule crops. Specifically, this study sought to answer the following question: to what extent do guayule crops attract native insect species that may be considered integrated into this novel ecosystem? What is the temporal dynamics of insect populations in regard to the phenological stages of the plant (specifically, the period between April and July)? Which insect taxa may pose potential risks as pests, according to bibliographical sources?

## 2. Materials and Methods

### 2.1. Study Area

Fieldwork was conducted over a 4-month period, from 25th April to 18th July, 2023, in Santa Cruz de la Zarza, a village belonging to the province of Toledo, in Central Spain. The cultivation area is surrounded by plots with herbaceous vegetation, primarily cereal crops. The climate is Mediterranean, characterized by the absence of rainfall during most of the year, hot summers (reaching over 40 °C), and relatively mild winters, with occasional night frosts reaching −5 °C in late winter. A weather station was installed within the study area to record temperature and rainfall.

### 2.2. Design and Settings

**Figure 1:**
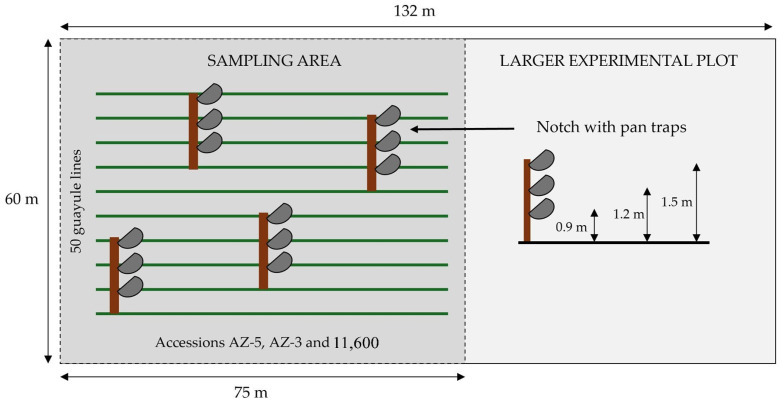
The schematic design of the trial.

A 75 m × 60 m sampling area was delineated within a larger experimental plot (132 m × 60 m) located at coordinates 39° 57′ 34.9200′′ N, 3° 10′ 18.1560′′ W, with an average altitude of 775 m above sea level. Within this sampling, four 1.5 m wooden posts were strategically placed. Each post had notches at heights of 0.9 m, 1.0 m, 1.2 m, and 1.5 m to accommodate pan traps (Figure 1). The traps were always positioned above the level of the growing plant, with their heights adjusted as the plant matured. No distinctions were made between the three guayule accessions (AZ-5, AZ-3, and 11,600) established in the 50 planted lines within the sampling area.

**Figure 2:**
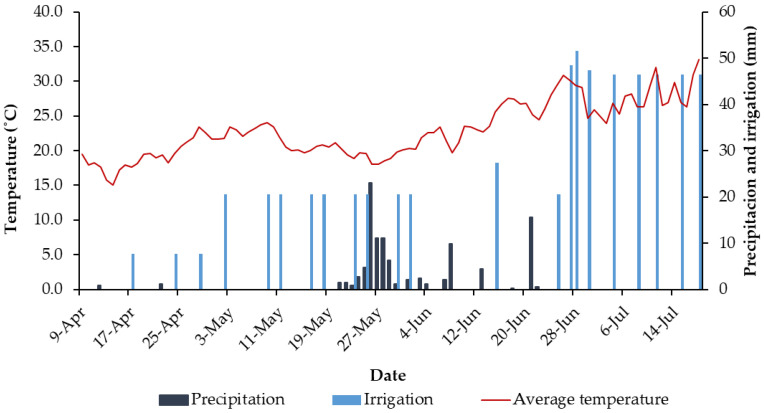
Rainfall, irrigation, and temperature in the experimental plot during the sampling period.

The plants were irrigated throughout this study in accordance with standard practices for this experimental plot, facilitating natural phenological changes. The level of irrigation and the temperature and rainfall data provided by the weather station are shown in Figure 2. This allowed for the observation of plant transformation, from an initial stressed state with dehydrated and silvery leaves, and complete lack of flowering, to a vibrant state with bright green leaves and developing flower buds. Throughout the sampling period, a representative, randomly selected, easily accessible group of plants (*n* = 11) was monitored to track flowering progress. Plants were photographed at each sampling session, and the number and maturity stage of its flowers were recorded.

### 2.3. Sampling of Insects

Twelve sampling sessions were conducted over the 4-month period on the following days: 25 April, 4 May, 10 May, 17 May, 25 May, 6 June, 14 June, 21 June, 28 June, 5 July, 13 July, and 18 July. Each sampling session lasted 4 h, from 9:00 a.m. to 1:00 p.m. Two methods were employed for insect sampling. The first method involved using a butterfly net to capture the insects observed on the top of the plant, but also on the branches, leaves, and flowers. This method allowed for the collection of insects interacting with flowers, for example, pollinators and herbivores. Sweeping was conducted throughout the delineated area, alternating between rows to ensure thorough coverage, to additionally capture sap-sucking insects and predators. Captured insects were placed in sealed plastic bags and stored in a refrigerated box until the end of the day. Insects that could not be captured were observed and recorded, mainly using voice notes.

The second method of sampling was conducted with pan traps at four equidistant points, each located 5 m from the boundary lines of the sampling area. Each pan trap had a volume of 500 mL containing soapy water. Pan traps were set up at 8:45 a.m. and collected at 1:15 p.m. Captured insects were collected by passing through a 0.1 mm double mesh filter (by folding) to separate the insects, which were finally stored in a jar.

All captured insects were transported to the laboratory. The subsequent treatment varied depending on the timing of processing. For immediate processing, the insects were first neutralized with ethyl acetate vapor. A small bottle with 1 mL of ethyl acetate in absorbent cotton was placed inside the bag with the specimens for 50 min. Insects were then pinned with entomological pins. Smaller or delicate insects were mounted before pinning. For insects captured by pan traps, sufficient drying time was allowed before pinning. If immediate processing was not feasible, the specimens were frozen to prevent decomposition and hardening of the exoskeletons, which would hinder the subsequent fixation processes. Once thawed, the same steps as described for immediate processing were followed.

Insects were stored in entomological boxes, separated based on their capture method (pan trap or net) and taxonomic order: Odonata, Dermaptera, Blattaria, Diptera, Hymenoptera, Coleoptera, Hemiptera, Lepidoptera, Orthoptera, Thysanoptera, Neuroptera, and Mantodea. Boxes were classified chronologically, typically one box per sampling session.

### 2.4. Identification and Trophic Guild Assignation

Insects were identified to species level whenever possible, with the help of specialists (see Acknowledgments) and field guides [24,25]. Otherwise, difficult or cryptic species were identified as morphospecies. Guild assignment was performed according to a large series of papers, but basically [24,25,26,27].

## 3. Results

### 3.1. Morphological Characterization

**Figure 3:**
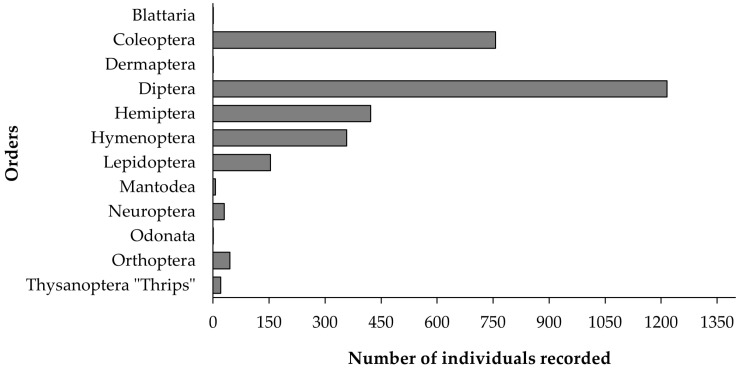
The number of total insects in the sampling period recorded by order.

A total of 3024 insects were counted through a combination of captures and observations. Identification was conducted at the morphospecies level when more precise identification was hindered by factors such as sexual dimorphism, phenotypic variability, or morphological similarity between species. Detailed identification data are provided in Supplementary Table S1. Insect sampling revealed the presence of up to 12 different orders between April and July. However, several orders were sparsely represented, with only one specimen each for Odonata, Blattaria, and Dermaptera, and six for Mantodea (Figure 3). Diptera was the most abundant order, followed by Coleoptera, Hemiptera, and Hymenoptera.

**Figure 4:**
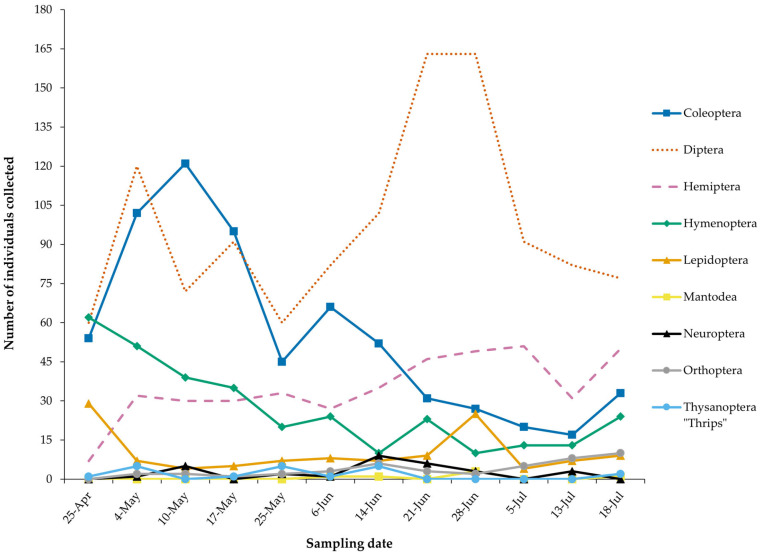
The temporal succession of insect abundance collected within the sampling period, grouped by orders. The orders Blattaria, Dermaptera, and Odonata are not included in the figure, as only one individual was found throughout the trial (25 May, 25 May, and 21 June, respectively).

Species richness varied across different insect orders throughout the sample period (Figure 4), although the number of insects collected did not appear to be significantly influenced by climatic conditions (compare Figure 2 and Figure 4). Populations of Coleoptera and Hymenoptera declined as the season progressed from spring to summer. Contrastingly, other notable orders such as Hemiptera and Lepidoptera maintained relatively stable populations with only minor fluctuations. Diptera populations experienced a significant increase in the second half of June, before returning to previous levels by the end of the sampling period.

**Figure 5:**
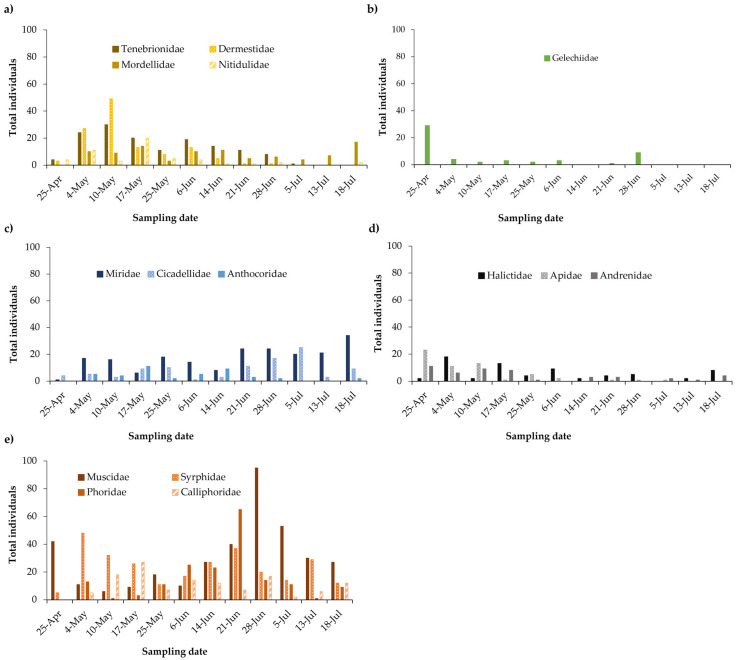
Distributions of abundance by family and time of the five most abundant orders: (**a**) Coleoptera, (**b**) Lepidoptera, (**c**) Hemiptera, (**d**) Hymenoptera, and (**e**) Diptera.

A more detailed examination of the family-level distribution within orders (Figure 5) revealed that Muscidae (325 individual specimens), Syrphidae (283), Phoridae (184), Calliphoridae (164), and Drosophilidae (84) were the most abundant families of Diptera.

Of the Muscidae family, *Musca domestica* (housefly) was the most commonly identified species, accounting for 252 of the 325 specimens. Nine additional Muscidae species were also identified. Of the Syrphidae family, *Eristalis tenax*, *Sphaerophoria scripta*, and *Syritta pipiens* were the most frequently encountered species. Analysis of the Phoridae family revealed up to seven possible different species with peak activity in June. Calliphoridae, primarily represented by the genus *Lucilia*, were consistently present throughout the sampling period, especially *Lucilia sericata*, reaching a peak of 105 insects from May to the end of the survey. Less frequent species from other families were also observed. Drosophilidae was consistently present throughout the sampling period, with daily counts generally not exceeding 10 per day.

Within the order Coleoptera (beetles), Dermestidae, Mordellidae, Chrysomelidae, and Tenebrionidae were the most abundant families. Dermestidae, particularly *Anthrenus* species, were present from the start of the sampling period until the end of June, disappearing in July. The Mordellidae family was one of the few families of the order Coleoptera that did not decline with the onset of summer; in fact, activity increased towards the end of the sampling period. Chrysomelidae, including *Cryptocephalus rugicollis*, and species of the subfamily Bruchinae or the tribe Alticini were consistently present but in low numbers until July, when their numbers decreased. Of the Tenebrionidae family, *Heliotaurus ruficollis* was very abundant on flowers in May, with peak counts reaching 30 in one sampling (May 10). This species was regularly observed until the end of June and disappeared in July.

Within the order Hemiptera (true bugs), three families were particularly prevalent: Miridae, Cicadellidae, and Anthocoridae. Miridae was the most abundant family (201 specimens categorized into 15 morphospecies). Its prevalence increased throughout spring, particularly within the genus *Lygus*. Cicadellidae (represented by 81 specimens, 27 belonging to the genus *Empoasca*, and the remaining corresponding to 5 other morphospecies) showed considerable abundance on 28 June and 5 July, with subsequent daily counts averaging 5 specimens. For Anthocoridae, (44 specimens, 3 species), *Orius laevigatus* dominated with 40 counts from May to June, and an additional specimen on 18th July.

For Hymenoptera (sawflies, wasps, bees, and ants), diversity was relatively low, with fewer than 50 species identified. The most abundant families were Halictidae, Apidae, and Andrenidae, all belonging to the superfamily Apoidea. Halictidae comprised 72 specimens, 47 of them belonging to the genus *Lasioglossum*. The peak abundance occurred in May, and declined in subsequent samplings, with a small rebound in the last sampling. Apidae, with 71 specimens, included the tribe Anthophorini (primarily at the end of April and beginning of May), and the genera *Ceratina, Eucera*, and *Thyreus*. Finally, one specimen of *Nomada basalis* and one of *Xylocopa violacea* were found. For the family Andrenidae, 58 specimens were found, mainly of the genus Andrena with 38 specimens. Andrenidae captures and observations drop drastically from 17 May onwards.

### 3.2. Accumulated Species Richness

**Figure 6:**
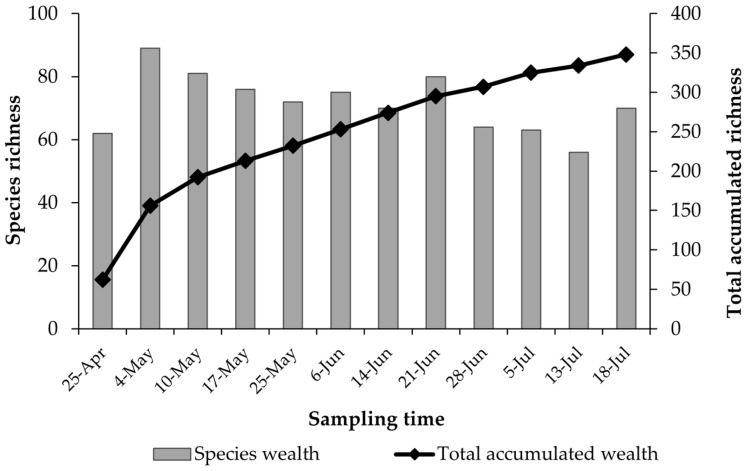
Species richness per sampling and total accumulated richness across the 12 samplings between April and July 2023.

The number of species recorded fluctuated across sampling sessions, peaking on 4th May (*n* = 89) and reaching a low on 13th July (*n* = 56) (Figure 6). The cumulative species richness curve continued to increase throughout the sampling period, failing to plateau at its endpoint of 352 species/morphospecies.

### 3.3. Plant Phenology

**Figure 7:**
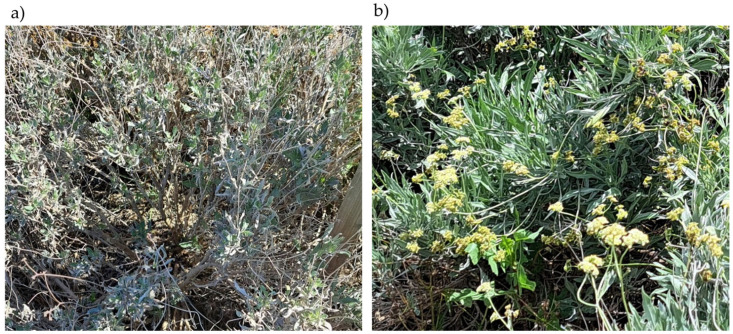
The status of the reference plant (**a**) at the beginning of the study, April 2023, and (**b**) at the end of the study, June 2023.

A phenological study of the plant was also conducted to better understand the pollination process and investigate more effective strategies to produce a larger number of fertile seeds as many of the insects sampled were observed visiting the guayule flowers, suggesting that they are potential pollinators of the plant. At the outset of the study in April 2023, the plants had not yet flowered. They had been left during the winter under natural conditions, resulting in a characteristic ashy appearance of their leaves and branches (Figure 7a). Regular watering was initiated to stimulate growth, and the plants reached peak flowering during the sampling session (Figure 7b).

**Figure 8:**
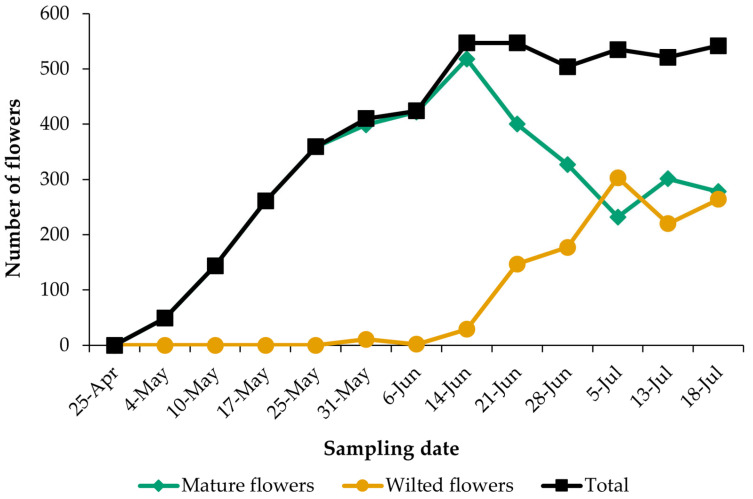
Floral phenology of the control plants in the plot in which flowering was monitored.

Figure 8 illustrates the evolution of plant flowering during the sampling period, differentiating between mature and wilted flowers. Plants under observation reached peak flowering on 13th June. Thereafter, the total flower count stabilized, although the number of mature flowers progressively declined while the number of wilted flowers increased. By 4th July, both types of flowers were roughly equal. Plants receiving limited irrigation continued to exhibit minimal flowering into mid-July, even as temperatures peaked in this region.

### 3.4. Trophic Role of the Insects Found

**Figure 9:**
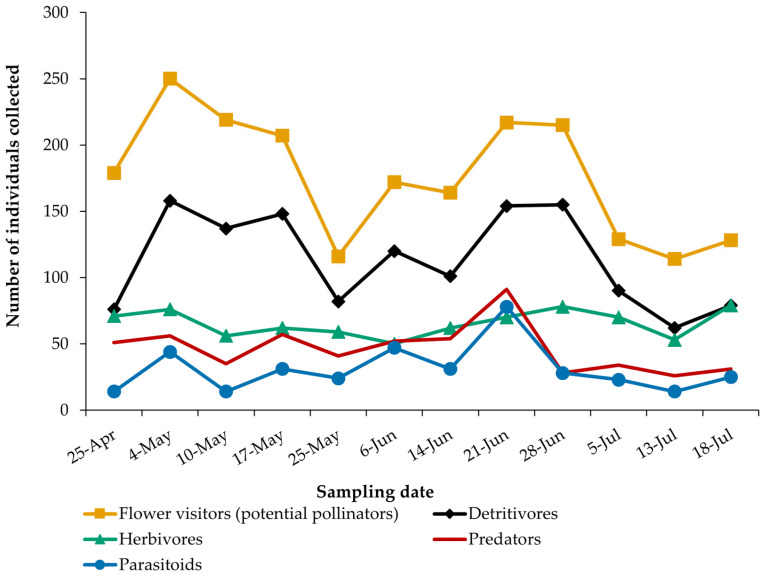
The number of individuals of each trophic guild during the sampling period.

Within the guayule cultivation area, various insect species likely play distinct trophic roles, forming complex networks of interactions with the plant. Some species are beneficial to guayule, while others can be harmful. Potential plant–insect interactions were analyzed in detail, and the trophic roles of more than 3000 captured specimens are shown in Supplementary Table S2 and summarized in Figure 9.

The peak number of insect visitors coincided with the early flowering stage of the plant, well before it reached its maximum bloom (compare Figure 9 with Figure 8). This suggests a potential mismatch between the timing of insect activity and the peak flowering period of the plant. The orders, genera, and species with the greatest pollination capacity are detailed below.

As seen in Supplementary Tables S1 and S2, the majority of the sampled Diptera species have a role in pollination (84 out of 86 species), particularly syrphids. Among these, *Eristalis tenax* was observed from May to mid-July. Its high pilosity enhances its pollination efficiency compared with other species, such as the family Calliphoridae. The genus *Lucilia* frequently interacted with flowers; however, their pollination efficiency is limited by their lack of specialized body parts for pollen transport. By contrast, the muscid *Musca domestica*, found in all samplings, is well equipped to pollinate flowers as it gathers nectar for consumption. Among Coleoptera, numerous species contribute to pollination (44 out of 79 species). For instance, *Tropinota hirta* and *Oxythyrea funesta* from the *Scarabaeidae* family have a lot of hair covering their bodies for pollen capture, surpassing the more frequently observed *Heliotaurus ruficollis* (highest number of annotations in the sampling) from the Tenebrionoidae family. Among Hymenoptera, members of the superfamily Apoidea were also common floral visitors, including Halictidae, Andrenidae, and Apidae (201 total specimens). While not as frequent as Diptera (1171) or Coleoptera (495) in guayule fields, their presence is always associated with pollination. Among Halictidae, the genus *Lasioglossum* was frequently found on mature flowers. For Apidae, members of this family are floral visitors; for example, the genus *Eucera* is a well-known generalist pollinator. In fact, most families within this order, to varying degrees, visit flowers. All Lepidoptera species are pollinators in their adult stage. Both diurnal and nocturnal pollinators were observed during the trial. Common diurnal pollinators included *Pieris rapae* (Pieridae), *Pyronia* spp. (Nymphalidae), and *Aricia cramera* (Lycaenidae), which were more prevalent in the latter half of this study. Nocturnal pollinators, observed while resting, primarily comprised species from the Gelechiidae family (more numerous at the beginning of the trial), and *Acontia lucida*, *Emmelia trabealis,* and *Tyta luctuosa* (Noctuidae), which were more prevalent in the middle phase of the study (Supplementary Tables S1 and S2).

However, other insects feed on plant tissue or sap. A large proportion of Hemiptera (47 of 53 species) are sap-suckers, including the families Miridae, Aphididae (aphids), Cicadellidae, and Issidae, represented by numerous species. This highlights the extent to which Hemiptera species exploit the plant, although their total abundance (386 total specimens) was lower than that of pollinators (2149 total specimens). Additionally, defoliators such as the Orthoptera (43 in total) were observed. At the beginning of the sampling, isolated specimens of *Anacridium aegyptium* (locusts) appeared, followed by other acridids from the genus *Oedipoda* (*O. charpentieri*, *O. caerulescens*) and the genus *Calliptamus* (*C. italicus*, *C. wattenwylianus*) as temperatures increased and summer approached. Lepidopteran caterpillars (158 specimens), such as *Tyta luctuosa* larvae or the Gelechiidae species, feed on the host plants. Certain Coleoptera, particularly Buprestidae (5 total specimens), exhibit phytophagous behavior, with adults feeding on the bark and larvae feeding on roots (Supplementary Tables S1 and S2). The large contrast between pollinators and herbivores is remarkable because it may reflect low herbivore levels or, alternatively, sampling bias towards specific herbivore groups; this issue will have to be addressed later.

The insect community also included predators (697 total specimens) that prey on other insects interacting with the plant. Camouflaged predators such as Mantids and *Chrysopa*, *Orius laevigatus* feeding on other Hemiptera, and ladybugs (*Coccinella septempuctata* and *Adalia* spp.) hunting aphids were observed. Parasitoids (402 total specimens) from various families of Hymenoptera (Ichneumonidae, Braconidae, Gasteruptiidae, Chrysididae, Aphelinidae, Evaniidae, Trichogrammatidae, Halictidae, and Apidae: genera *Nomada* and *Thyreus*), Coleoptera (Cleridae), and Diptera (Phoridae, Sarcophagidae, Conopidae, Pipunculidae, and Tachinidae) were also present (Supplementary Tables S1 and S2).

Detritivores (1148 total specimens) exhibited a similar pattern to pollinators, as many species fulfill both roles, but were found in lower numbers. Herbivores, although present, remained below 100 in the samplings, and showed no significant fluctuation with plant development. Predators and parasitoid organisms followed a similar trend, both peaking on June 21st and gradually declining thereafter (Supplementary Tables S1 and S2); this is consistent with xeric, limiting conditions of Mediterranean summer, which reduce prey availability in many instances [28].

## 4. Discussion

This study is the first of its kind conducted in Europe. Indeed, Europe has only a few plots dedicated to guayule, including a cluster of seven in the vicinity of Santa Cruz de la Zarza, Toledo, where this study was undertaken. Consequently, most reports dealing with guayule insect loads and interactions originate from the USA. Many of these reports date back to the first half of the 20th century, focusing on insect pests that attacked young plants in greenhouses. This is because, for many years, the primary propagation method involved transplanting seedlings rather than direct planting. This study was performed with a broader approach, with the aim of discovering all the interactions, both beneficial and detrimental, between entomological fauna and guayule outside its natural habitat. Although a large number of insects were counted (3024 distributed in 12 different orders), the accumulated richness in the sampling period did not reach a plateau, suggesting that the sampling period was insufficient in fully capturing the species diversity at the site. Extending the sampling period until September or October, when many herbivore species in Mediterranean habitats typically rebound [28,29], would probably be necessary.

The process of local fauna acclimatation to a new species can be lengthy [30,31,32]. As the guayule crop at Santa Cruz de la Zarza is relatively new, having been established for about a decade, many insect species may still have limited interactions with it. Some species, even those found in nearby crops, may not be effective pollinators of guayule. Herbivores, in particular, may be negatively impacted by unknown, potentially harmful compounds (allelochemicals) that they cannot detoxify [30,32]. Consequently, the number of such species may not increase as rapidly as in other recently established crops in the area. Guayule resin, a complex mixture of secondary metabolites, is very rich in terpenoids, which have been traditionally related to the natural resistance of guayule against pests [33,34]. Among the many terpenoids in the guayule resin, guayulins remain of particular importance, as they have been linked to insect-deterrent activity [7,35]. Although the present study did not test this hypothesis directly, the overall low abundance and diversity of herbivores observed are consistent with the presence of such chemical defenses. In comparison, other non-native crops commonly cultivated in the region, such as sunflower or maize, tend to be colonized more rapidly by herbivorous insects, suggesting that guayule’s distinctive phytochemical profile may act as an effective ecological barrier during its early establishment. However, as the crop becomes more established, the risk of herbivore recruitment is expected to increase [31,36], suggesting a potential shift in insect community composition over time.

The most comparable study was conducted by [9], which also involved insect sampling of guayule fields, but using an insect vacuum. Despite significant differences in climate, soil, cultivation methods, plant–insect adaptation time, and sampling techniques, some general comparisons can be drawn. The successful industrial cultivation of guayule requires a comprehensive approach that addresses various factors, such as production optimization, including mechanized planting and harvesting, and efficient rubber extraction techniques; agronomic management, including effective cultivation practices to maximize yield and quality; acclimatization, adapting the plant to diverse growing environments; and environmental sustainability, minimizing potential environmental impacts. The historical cultivation of guayule in Spain between 1940 and 1960 demonstrated its stability as a crop and its low invasive potential [4]. Despite the cultivation of nearly 4000 hectares during this period [5], very few isolated plants remain today. To assess the potential environmental impact of reintroducing guayule, it is crucial to study its interaction with native insect fauna.

The scientific understanding of the arthropod communities associated with cultivated guayule, particularly outside its native range, is presented in Table 1, organized by taxonomic order and family to allow comparison of the insect species reported across the different studies.

Insects that prey on other insects accounted for 36% in the American study, nearly double that found in the present study (19%). Specimens belonging to the family Anthocoridae (Hemiptera), more specifically to the genus *Orius*, were common in both locations. *Orius tristicolor* was prevalent in the subdesert ecosystems, while *Orius laevigatus* was common in the Mediterranean. Additionally, both studies identified the genus *Chrysopa* (order Neuroptera), and the family Melyridae and Coccinellidae (order Coleoptera).

In the plants sampled by [9], other commonly found predators included Hemiptera (*Nabis alternatus* and *Geocoris pallens*), Neuroptera (*Micromus subanticus*), and Coleoptera (*Collops vittatus*) (Table 1). In Spain, the most common predators were *Sphaerophoria scripta*, whose larvae are voracious aphid eaters. Some species of the Phoridae family also exhibit this behavior in their larval stage, as do Staphylinidae, and the previously mentioned *Orius laevigatus*, *Chrysopa* sp. and *Coccinella septempunctata*.

Parasitoids and detritivores made up 6% and 1%, respectively, in Texas, compared with 21.6% and 25.6% in the present study (Table 1). In the fields of El Paso and Pecos (Texas), the primary parasitoid was *Aphidius matricariae*, belonging to the family Braconidae. By contrast, a diverse parasitoid community was found in Santa Cruz de la Zarza, including families such as Halictidae, Gasteruptiidae, Trichogrammatidae, Aphelinidae, and Ichneumonidae (Hymenoptera), and Phoridae, Sarcophagidae, Conopidae, Pipunculidae and Tachinidae (Diptera). The substantial disparity in detritivore abundance between the two regions may be attributed to the contrasting habitats; the Mediterranean climate promotes greater organic matter production than the arid conditions in the southern part of North America. But this may also be partially attributed to sampling properties, since [9] used vacuum sampling and did not specifically focus on pollinators.

The study by [11] identified several potential pests that could harm guayule plants in Zacatecas, Mexico, including *Ceroputo yuccae*, *Orthezia* sp., and *Targionia dearnessi* (Hemiptera), and *Pityophthorus nigricans* (Coleoptera) (Table 1). However, these specific species or their equivalents were not observed in the present study. Romney (1946) [12] expanded the research to several regions of Northern Mexico, and discovered additional potential pests, including some that affect the roots of guayule, such as *Targionia yuccarum* (Hemiptera: Diaspididae). This species was not found in the present study, because the lower part of the plant was not sampled. Similarly, *Lecanodiaspis* sp. (Hemiptera: Lecanodiaspididae) and *Tachardiella cornuta* (Hemiptera: Kerriidae), which were found on branches in Mexico, may have been overlooked in Spain due to sampling limitations. Most beetles observed were in their larval stage, including species from the genera *Urilleta*, *Chalcophora*, and *Dicerca* [12]. The latter two belong to the Buprestidae family, which also includes the genera *Acmaeodera* and *Anthaxia*, found in the experimental plot field in Santa Cruz de la Zarza. Moths (*Eucosma*, *Tortricidae*) and mealybugs (Pseudococcini, Pseudococcidae) were also present, but no specific species from these families were reported in the present study. By contrast, grasshoppers (species of the family Acrididae), bugs (*Lygus*, Miridae), and leafhoppers (*Empoasca*, Cicadellidae) found in the present study in Spain do have counterparts in Mexico. Romney highlighted that most species found were more prevalent on naturally occurring plants, while those on planted species had a lower incidence of potential pests. When comparing the data of the present study with that of Refs. [9,11], species-level overlaps were absent, but taxonomic similarity remained. However, the present study did share the families Miridae (especially the genus *Lygus*) and Cicadellidae with the Romney study.

Similar species to those identified in the study by [8] were observed in the present study. For example, several chrysomelids (Coleoptera) could have a similar impact to *Diabrotica undecimpunctata,* for example, *Cryptocephalus rugicollis*, which feeds on leaves and flowers of Asteraceae. While it was not possible to determine if some of the mirids found belong to the genus *Lygus* (see Supplementary Table S1), all of them likely share the same feeding behavior, sucking the sap from the plants they inhabit.

It is important to consider the effects that the genus *Lygus* could have on guayule seedlings, their development, and seed production [10,12,16,20]. Romney (1946) [12] demonstrated that *Lygus* nymphs feeding on young guayule plants can delay seed formation, growth, and viability, as well as overall plant development. In severe cases, *Lygus* nymphs were observed to consume entire seeds. The influence of pollinating insects on these plants was not investigated in these studies, as the primary focus was on the potential harm caused by *Lygus* species and their accurate taxonomic identification. Cassidy (1950) [10] further noted feeding preferences among different *Lygus* species: *L. hesperus* and *L. elisus* tended to target seeds while *L. sallei* preferred growing tips. These observations were later corroborated by [20], who noted that *L. hesperus* preferred reproductive tissues. Addicott and Romney (1950) [16] experimentally exposed *Lygus hesperus* adults to young guayule plants, recording increased damage to branch tips and wilting leaves.

*Lygus* species, known to feed on guayule plants, have been a target for pest management strategies. Early efforts involved the use of experimental arsenical insecticides [22]. More recent research has focused on biological control methods, such as identifying and characterizing natural enemies, including *Anaphes ovijentatus*, *Leiphoron uniformis,* and *Alophorella* spp. [21].

The most comprehensive study on herbivores affecting guayule [10] lists insects common to those found in Santa Cruz de la Zarza, including grasshoppers (Acrididae); thrips (Thysanoptera); the families Aphididae, Miridae, and Cicadellidae (Hemiptera); Formicidae (Hymenoptera); and Elateridae, Tenebrionidae, and Chrysomelidae (Coleoptera). Other insects including termites and flies (*Agromyza virens*), moth larvae, and crickets were found in the same study. Essig (1944) [13] reported a new aphid species, *Cerosipha californica*, which feeds on roots. Several important herbivore species of the Aphididae family were recorded in the experimental plot samplings, although the new species described by Essig was not, as the root system was not examined in the present study. Research on moth caterpillars has focused on larvae of the genus *Spodoptera* [6,37] and *Heliothis zea* larvae [6]. The latter study investigated artificial diets based on extracts of different *Parthenium* species, finding that *P. tomentosum* and *P. schottii* more strongly inhibited larval development than *P. argentatum*. Céspedes et al. (2001) [37] tested several guayule-specific compounds and found inhibition and even mortality in *Spodoptera frugiperda* larvae. However, specimens from these genera were found in the samplings in Santa Cruz de la Zarza.

Despite the contrast between the arid American habitat and the Mediterranean climate, the shared family composition and functional roles suggest similar trophic structures in both sites. However, species richness is expected to increase rapidly in the Spanish site during future years, mirroring the trend observed in the introduced plants [38,39].

It should be noted that the literature does not provide a comprehensive overview of all insect species interacting with guayule in its natural habitat. Most studies focus on a specific type of interaction, typically insect herbivory and its negative impact. Other aspects, such as the beneficial effects of pollinating insects, are often overlooked. The most comprehensive study to date was reported by [9]; however, even with this information, it cannot be definitively determined whether one habitat supports a greater diversity of species than the other.

While it is intriguing to consider that the negative impact of certain insects could be offset by beneficial ones, conclusive evidence is still lacking. In the present study in Santa Cruz de la Zarza, the number of possible pollinators found was much higher (2249) than the number of herbivores (874). Among the herbivores, Miridae, particularly the *Lygus* genus, accounted for nearly 23% of the total, highlighting their potential importance. Both samplings in Santa Cruz de la Zarza, the preliminary one [23] and the present study, represent a total of 7 months across a period of 2 years of cultivation. Further studies are needed to understand the complex interactions between species in this Mediterranean region and their specific impact on guayule. Research on insect-mediated herbivory and its effects on guayule growth and defense strategies should be prioritized, as well as mapping the seasonal and diurnal dynamics of insect groups, including nocturnal sampling to capture under-represented nocturnal activity. Long-term studies are also essential to detect inter-annual variability and to understand how these interactions respond to environmental fluctuations and broader ecological changes. These efforts will contribute to a deeper understanding of the ecological drivers of guayule populations and help clarify the role of biotic interactions in their adjustment and productivity under Mediterranean environmental conditions. In particular, it may contribute to explaining the consequences of the observed mismatch between insect visitation rates and maximum flowering of the plants, since if it is a common phenomenon, it could have ecological consequences such as a reduction in pollination efficiency and seed production. Also, the remarkable contrast between pollinators and herbivores may be clarified; it may reflect low herbivore levels or, alternatively, sampling bias towards specific herbivore groups.

## 5. Conclusions

A remarkable diversity of insect species, spanning up to 12 different orders, was observed in this young, non-native crop. While numerous potential pollinators were identified, their effectiveness in contributing to seed production remains uncertain and requires further investigation. Specific studies on some of the pollinators found in greater numbers in the present study (e.g., Diptera, specifically syrphids) could provide valuable insights into seed production. In this context, future research will include pollination efficiency trials to assess the specific contribution of syrphid flies to guayule reproduction. The presence of several herbivorous insects, such as defoliating orthopterans (grasshoppers), which are very common in the fields of other nearby crops, and sap-sucking Hemiptera (aphids, mirids, and others), was noted. However, their current populations are likely not sufficient to pose an immediate threat to the crop. It is possible that the production of insect-toxic compounds by this plant may deter certain pests that have yet to adapt, but it is to be expected that as the crop becomes more established, the risk of herbivore recruitment increases [31,36]. On a more encouraging note, many natural enemies of these sucking insects were found, including predatory insects (Anthocoridae, Reduvidae, some Coleoptera such as Coccinellidae), as well as parasitoids of different orders and families. These beneficial organisms may help control pest populations and mitigate potential damage. Currently, there are no immediate signs of pest outbreaks. As the crop continues to mature and integrate into the local ecosystem, it is likely that insect interactions, both beneficial and detrimental, will evolve over time.

## Figures and Tables

**Table 1:** Data from different countries [9,11,12,23] were organized into matching families and orders for different species.

Order	Family	Mexico [11]	Mexico [12]	USA [9]	Spain [23]
Coleoptera	Anthicidae			*Notoxus calcaratus*	Unidentified
	Buprestidae		*Chalcophora* sp. *Dicerca* sp.		*Anthaxia* spp. *Acmaeodera* spp.
	Chrysomellidae			*Diabrotica undecimpunctata* *Epitrix hirtipemis*	*Cryptocephalus rugicollis*
	Coccinellidae			*Hippodamia convergens*	*Coccinella septempunctata* *Adalia* spp.
	Curculionidae	*Pityophtorus nigricans*	*Pityophtorus mexicanus*		*Cleopomarius* sp.
	Meloidae			*Epicauta callosa*	*Cerocoma* sp.
	Melyridae			*Collops vittatus*	*Malachius bipustulatus Enicopus* sp.
	Mordellidae			*Mordellistena* sp.	Unidentified
	Ptinidae (Anobiinae)		*Urilleta* sp.		
Hemiptera	Anthocoridae			*Orius tristicolor*	*Orius laevigatus*
	Cicadellidae		*Empoasca* spp. *Cloanthanus heldoranus*	*Empoasca* spp.	*Empoasca* spp.
	Diaspididae	*Targionia dearnessi*	*Targionia yuccarum*		
	Geocoridae			*Geocoris pallens*	
	Issidae		*Hysteropterum sepulchralis*		Unidentified
	Kerriidae		*Tachardiella cornuta*		
	Lecanodiaspididae		*Lecanodiapsis* sp.		
	Lygaeidae			*Nysius* sp. *Xyonysius californicus*	*Spilosthetus pandurus*
	Membracidae			*Spissistulus festinus*	Unidentified
	Miridae		*Lygus oblineatus* *Polymerus basilis*	*Lygus hesperus* *Lygus lineolaris*	*Lygus* spp.
	Nabidae			*Nabis alternatus*	
	Ortheziidae	*Orthezia* sp.			
	Pseudococcidae	*Ceroputo yuccae*	*Pseudococcini*		
	Rhopalidae			*Hormostes reflexulus*	Unidentified
	Tingidae			*Corythucha morrilli*	
Hymenoptera	Braconidae			*Aphidius matricariae*	Unidentified
	Formicidae			Unidentified	*Camponotus*
Lepidoptera	Tortricidae		*Eucosma*		
Neuroptera	Chrysopidae			*Chrysopa* spp.	*Chrysopa* sp.
	Hemerobiidae			*Micromus subanticus*	
Orthoptera	Acrididae		*Encoptolophus pallidus* *Trachyrhachys kiowa* *Platylactisca azteca*	*Trimerotropis pallidipennis* (occasional)	*Calliptamus* spp.
				*Derotmena haydenii* (rare)	

## Data Availability

The data presented in this study are available upon request from the authors.

## Supplementary Materials

The following supporting information can be downloaded at https://www.mdpi.com/article/10.3390/insects16080808/s1, Table S1: Identification; Table S2: Functions.
